# Cognitive Sequelae of Silent Ischemic Lesions Following Carotid Artery Stenting: Possible Role of Age-Related Moderation

**DOI:** 10.3389/fnagi.2021.732617

**Published:** 2022-01-12

**Authors:** Kuo-Lun Huang, Meng-Yang Ho, Yeu-Jhy Chang, Chien-Hung Chang, Chi-Hung Liu, Hsiu-Chuan Wu, Ting-Yu Chang, Tsong-Hai Lee

**Affiliations:** ^1^Stroke Center and Department of Neurology, Linkou Chang Gung Memorial Hospital and College of Medicine, Chang Gung University, Taoyuan, Taiwan; ^2^Clinical Psychology Program, c/o Department of Occupational Therapy, Chang Gung University, Taoyuan, Taiwan

**Keywords:** carotid stenosis, carotid artery stenting, cognition, ischemia, degeneration, practice effect

## Abstract

**Background:** The occurrence of ischemic lesions is common in patients receiving carotid artery stenting (CAS), and most of them are clinically silent. However, few studies have directly addressed the cognitive sequelae of these procedure-related silent ischemic lesions (SILs).

**Objective:** In this study, we attempted to investigate the effects of SILs on cognition using a comprehensive battery of neuropsychological tests.

**Method:** Eighty-five patients with unilateral carotid stenosis and 25 age-matched healthy volunteers participated in this study. Brain MRI was performed within 1 week before and 1 week after CAS to monitor the occurrence of post-CAS SILs. A comprehensive battery tapping reading ability, verbal and non-verbal memory, visuospatial function, manual dexterity, executive function, and processing speed was administered 1 week before and 6 months after CAS. To control for practice effects on repeated cognitive testing, the reliable change index (RCI) derived from the healthy volunteers was used to determine the cognitive changes in patients with carotid stenosis.

**Results:** Among the 85 patients with carotid stenosis, 21 patients received medical treatment (MED group), and procedure-related SILs were noted in 17 patients (SIL+ group) but not observed in 47 patients (SIL– group) after undergoing CAS. Two-way (group × phase) ANOVA revealed that the volunteer group showed improved scores in most cognitive tests while only limited improvement was noted in the SIL– group. The MED and control groups tended to show improvement in the follow-up cognitive testing than the SIL+ group. However, most of the cognitive changes for each patient group did not exceed the upper or lower limits (*z* = ±1.0) of the RCI.

**Conclusions:** Although the occurrence of procedure-related SILs is common in patients undergoing CAS, their impacts on cognitive changes after CAS may be limited. The practice effect should be taken into consideration when interpreting cognitive changes following CAS.

## Introduction

Stroke is one of the leading causes of death in the world, and vascular cognitive impairment is the second most common cause of dementia (Madigan et al., 2016). Carotid stenosis is one of the major stroke risk factors, which can lead to cognitive impairment secondary to thromboembolism or hypoperfusion with insufficient collateral compensation (Sztriha et al., 2009; Wallin et al., 2018). Carotid endarterectomy (CEA) and carotid angioplasty and stenting (CAS) can be used for stroke prevention in patients with significant carotid stenosis (CS) (Brott et al., 2016). However, whether restoration of blood supply after carotid revascularization is beneficial to cognition remains inconclusive, and only 10–15% of patients presented with either cognitive improvement or decline after carotid revascularization procedure (Paraskevas et al., 2014). Many factors have been proposed to account for the inconsistent results of the potential beneficial effects of revascularization procedures on cognition, including the pre-existing infarcts, age-related cognitive declines and neurodegeneration, and peri-operation risks (Moroni et al., 2016; Porcu et al., 2020).

In addition to the peri-operation stroke risk, one of the hypothetical mechanisms of cognitive deficits produced by CEA or CAS is procedure-related silent ischemic lesions (SILs). Diffusion-weighted imaging (DWI) studies have revealed that the occurrence of SILs can be detected for up to 71% of patients undergoing CAS (Wasser et al., 2011). The clinical significance of SILs has been addressed (Kastrup et al., 2003), but relatively fewer studies have directly investigated their cognitive sequelae (Tiemann et al., 2009; Capoccia et al., 2010; Zhou et al., 2012; Laza et al., 2013). This highlights the need to examine the effects of SILs on cognition, because the presence of these lesions may not only confound the net differences in cognitive changes (improved or worse) following CAS or CEA, but also has important implications in the cost/effectiveness of CAS, which appears to be more vulnerable than CEA to produce SILs (Bonati et al., 2010).

In this study, we attempted to investigate the effects of SILs on a more comprehensive battery of neuropsychological tests by including a reference group of healthy subjects to derive the reliable change indices (RCIs) for determining whether there is any significant improvement or decline in any specific cognitive domains in patients undergoing CAS. Using RCIs in defining improvement or decline in cognitive functions has long been proposed to control for the practice effects and regression toward the mean of the testing scores, which have been commonly observed in neuropsychological literature, particularly when repeated testing of the same cognitive domains were involved (Tuokko and Smart, 2018). However, these phenomena or possibilities have rarely been addressed in the cognitive changes following carotid revascularization. We expected that applying the psychometrically defined thresholds might be of help to elucidate the effects of post-CAS SILs on cognition.

## Materials and Methods

### Participants

#### Patients

Eighty-five right-handed patients (81 males, 4 females) with carotid stenosis participated in this study. Their ages ranged between 49 and 83 (Mean ± SD = 66.1 ± 8.2) years old. They were all recruited from the Stroke Registry Dataset (Lee et al., 2011), Lin-Kou Chang Gung Memorial Hospital, and enrolled based on the following inclusion criteria: (1) the left or right internal carotid artery showed moderate to severe stenosis (≥50% by the NASCET criteria) on angiography; (2) age was ≥45 years; (3) the literacy level was at least equivalent to the level of the 4th-grade pupils on the Chinese Graded Word Reading Test (CGWRT) (Huang, 2001); (4) the score on the National Institutes of Health Stroke Scale (NIHSS) score was ≤ 8; (5) the score for the Barthel Index was ≥80. The exclusion criteria were: (1) the MMSE score ≤ 20; (2) the Clinical Dementia Rating Scale (CDR) ≥1; (3) the presence of any expressive/receptive language disturbance; (4) a history of coronary artery bypass surgery; and (5) the presence of severe renal disease (serum creatinine >3 mg/dl) or undergoing hemodialysis therapy.

#### Volunteers

Twenty-five age-matched volunteers were recruited by advertisements placed at the notice boards around the hospital to serve as the control group for examining the extent of practice effects produced by repeated testing. They had no history of head injuries, stroke, psychiatric illness, or significant carotid stenosis (>50%) based on the color-coded carotid duplex (CCCD) and magnetic resonance angiography (MRA). Their MMSE scores were all well above 25 points.

The study protocol and procedure for obtaining informed consent were complied with the Helsinki Declaration, and were approved by the Institutional Review Board of Chang Gung Memorial Hospital. All participants provided written informed consent.

### Treatment of Carotid Stenosis

Two experienced endovascular physicians performed the CAS procedures. Depending on patients' conditions, physicians decided whether or not the embolic protection devices (EPDs) needed to be applied on each individual. Post-stenting angioplasty with balloon dilation was done in all patients. All patients received intra-procedural heparinization. A combination of aspirin (100 mg/day) and clopidogrel (75 mg/day) was administered at least 72 h before stenting procedures. After stenting procedure, clopidogrel was prescribed for 3 months and aspirin was maintained indefinitely. Two independent neurologists with expertise in stroke evaluated the patients the day before and 24 h after the stenting procedures.

### MRI Studies

Brain MRI was performed within 1 week before and 1 week after CAS, and the scanning protocol has been described in previous studies (Huang et al., 2011, 2014a). In brief, anatomical MRI was obtained at a 1.5- or 3.0-Tesla scanner with 5-mm slice thickness and 0.5-mm inter-slice gap for all sequences, including axial T1-weighted, fluid-attenuated inversion recovery (FLAIR) and DWI sequences.

The baseline brain imaging characteristics were visually evaluated. The severity of periventricular and deep white matter leukoaraiosis was scored as follows: 0 = absence, 1 = focal lesions, 2 = beginning confluence of foci, or 3 = diffuse involvement of the entire region (Wahlund et al., 2001). The severity of cerebral infarct was visually rated as 0 = no lesion, 1 = one focal lesion (≥5 mm), 2 = more than one focal lesion, and 3 = confluent lesions (Lee et al., 2017). The severity of hippocampal atrophy was rated by the Schelten medial temporal lobe atrophy (MTA) score (Scheltens and van de Pol, 2012). Follow-up MRI was done within 1 week after carotid artery stenting to evaluate the occurrence of new ischemic lesions on DWI images that was not present on the pre-treatment images, and post-treatment SILs were those DWI lesions without corresponding focal neurological manifestation. Trained neurologists, blinded to clinical and cognitive conditions, conducted the qualitative, and quantitative measurements of MRI imaging as described in our previous study (Huang et al., 2014b).

### Neuropsychological Assessment

Patients underwent a comprehensive neuropsychological test battery 1 week before and 6 months after CAS. These tests were chosen because our previous studies have shown they were sensitive to the cognitive deficits in patients with carotid stenosis (Huang et al., 2014a, 2018). Global cognitive function was assessed with the MMSE and CGWRT, and the neuropsychological measures corresponding to each cognitive domain were summarized in the Supplementary Table 1.

### Data Analyses

The demographic and medical data of subjects at the enrollment were summarized by descriptive statistics with one-factor ANOVA or χ^2^-tests for group comparisons where appropriate.

#### T Score Transformation and Domain Assignment for Cognitive Results

More than 20 neuropsychological measures were recorded in this study. Some of these measures are purported to tap similar specific cognitive functions, thereby a scale-reduction procedure was applied to minimize the potential redundancy of multiple comparisons. Each raw score of the neuropsychological measure was transformed to T score (Mean = 50, SD = 10) based on the local normative data corresponding to each participant's age. After this, we adopted a data-driven approach to carry out a principal component analysis (PCA) on the T scores of all neuropsychological measures of 94 participants randomly selected from a previous study for determining the factor loadings of each measure on extracted latent components (cognitive domains). Varimax rotation of the components revealed 7 specific cognitive domains were extracted from this dataset, accounting for >72% of the total variance. Of note, the T score of the CGWRT was not included in the PCA for scale reduction, because it has commonly been used as a measure for estimating premorbid general ability (Chen et al., 2009). Except for the CGWRT, the T scores for cognitive measures corresponding to each component were averaged to derive the mean T score for each specific cognitive domain.

The average T scores for each cognitive domain as well as the T scores of the CGWRT obtained before and after treatment were analyzed by two-factor (group × phase) ANOVA with repeated measure on the latter factor, and *post-hoc* comparisons using Bonferroni corrections were applied where appropriate. Since cognitive changes after treatment can be affected by pre-existing cerebrovascular pathologies, age-related cognitive decline, and neurodegeneration, we further employed ANCOVA (analysis of covariance) with repeated measures to adjust for potential variables, such as demographic factors (age and education in Model I), neurodegeneration factor (MTA score in Model II), and vascular factors (severity of leukoaraiosis, infarct, and carotid stenosis in Model III).

#### Reliable Change Index

As the results of two-factor (group × phase) ANOVA indicated there was strong and significant main effect of phase on the T scores for many neuropsychological tests, suggesting that the possibility of practice effects brought about by repeated testing needs to be examined. The method proposed by Chelune et al. was used to derive the RCI based on control group's testing scores between the pre- and post-treatment phases that showed significant main effect of phase (Chelune et al., 1993). The RCIs for the testing scores were calculated based on the following equation:


$$\begin{array}{l} RCI=\left[\left(X_{2}-X_{1}\right)-\left(M_{2}-M_{1}\right)\right]/{\mathrm{SE}}_{D} \end{array}$$

where X_2_ = individual's post-treatment score, X_1_ = individual's pre-treatment score, *M*_2_ = mean post-treatment score and *M*_1_ = mean pre-treatment score of the control group, respectively. The correlation coefficients (*r*s) of the scores between two phases in the control group were calculated to derive the SEM of each test, where SEM = *SD*_pre−treatment_ × √(1–*r*); and *SE*_D_ for each test was determined, *SE*_D_ = √2 × (SEM)^2^. The cutoff values of +1.0 and −1.0 were used to indicate a reliable improvement and decline, respectively. The frequency and percentage of patients whose score changes fell in the significant ranges were calculated. χ^2^-tests were used to examine the differences in all patient groups.

## Results

### Demographic and Medical Data

We recruited 85 patients with carotid stenosis and 25 healthy volunteers in this study. After the treatment options were explained in details, 64 patients decided to undergo the CAS, whereas the remaining 21 patients chose to receive medication treatment and served as the medication (MED) group. Based on the follow-up MRI scanning, 17 of the 64 patients undergoing CAS had newly acquired asymptomatic DWI lesions after stenting; and they were allocated to the SIL+ group, whereas the remaining 47 CAS patients were assigned to the SIL– group.

Table 1 shows the pre-treatment demographic data and medical conditions of the volunteers and all patient groups. The ages, years of education, and baseline MMSE scores did not significantly differ between groups. The *post-hoc* analyses showed there was no significant difference in the severity of carotid stenosis, leukoaraiosis, infarct score, and MTA score among carotid stenosis patients, but the volunteers had less vascular and degeneration burdens than carotid stenosis patients.

**Table 1:** The demographic data and imaging characteristics among groups.

	**SIL– group**	**SIL+ group**	**MED group**	**Control group**	***p***
*n*	47	17	21	25	
**Demographic data**
Age, years	66.0 ± 8.8	68.5 ± 7.2	64.2 ± 7.4	64.9 ± 4.2	0.33
Years of education	9.2 ± 3.7	9.2 ± 3.6	9.0 ± 3.2	10.0 ± 2.8	0.78
MMSE	27.2 ± 2.1	27.1 ± 2.3	27.1 ± 1.8	27.3 ± 1.5	0.98
Male, *n* (%)	44 (94)	17 (100)	20 (95)	20 (80)	0.08
Hypertension, *n* (%)	41 (87)	17 (100)	16 (76)	8 (32)	<0.0001
Hyperlipidemia, *n* (%)	24 (51)	12 (71)	10 (48)	15 (60)	0.46
Diabetes mellitus, *n* (%)	16 (34)	10 (59)	11 (52)	1 (4)	<0.0001
EPD employment, *n* (%)	30 (64)	14 (82)	–	–	0.14
**Baseline imaging characteristics**
Ipsilateral stenosis, %	79.6 ± 13.1^†^	79.4 ± 10.3^†^	84.3 ± 19.6^†^	12.0 ± 9.7	<0.0001
Contralateral stenosis, %	41.4 ± 34.7^†^	24.9 ± 25.6	35.8 ± 22.3^†^	5.4 ± 7.2	<0.0001
Leukoaraiosis severity	1.1 ± 0.6^†^	1.4 ± 0.8^†^	1.0 ± 0.7^†^	0.5 ± 0.5	<0.0001
Infarct score	1.4 ± 1.0^†^	1.7 ± 1.2^†^	1.1 ± 1.2^†^	0.0 ± 0.0	<0.0001
Medial temporal atrophy score	0.7 ± 0.8	1.1 ± 0.8^†^	0.5 ± 0.6	0.3 ± 0.6	0.005

*CAS, carotid artery stenting; EPD, embolic protection device; MMSE, Mini Mental Status Examination; SIL–, absence of new silent ischemic lesions after stenting; SIL+, presence of new silent ischemic lesions after stenting;† p < 0.05 compared to the control group*.

### Cognitive Performance

In patients with carotid stenosis, baseline cognitive function was mostly correlated with education as well as the degeneration severity (the MTA score), while it was less associated with the severity of ipsilateral carotid stenosis, infarct score, and leukoaraiosis (Supplementary Table 2). Table 2 shows the averaged (±SD) T scores of the specific cognitive domains obtained from the pre- and post-treatment phases in all groups, and the results of main effects of two-factor (group × phase) ANOVA. The main effects of group were significant in most mean T scores of cognitive domains. *Post-hoc* comparisons revealed that the cognitive performance of volunteers was significantly better than that of all patient groups in both phases, respectively. However, the mean T scores of all specific cognitive domains between patient groups did not significantly differ in either phase. For the main effect of phase, the inter-phase cognitive improvement was observed in most tests for the volunteers, but it was only noted in a few tests for each patient group. Similar trends were observed after adjustment for potential confounding factors in the ANCOVA of Models I–III (Tables 3–5). In addition, the vascular-related imaging markers were only correlated with the changes in executive function after carotid artery stenting, while MTA score was correlated with changes in visual memory and executive function (Supplementary Table 3).

**Table 2:** Comparisons of cognitive domain performance before and after treatment by ANOVA.

**Group**	**Pre-treatment phase**				**Post-treatment phase**				***F***		
	**DWI(–)**	**DWI(+)**	**MED**	**Control**	**DWI(–)**	**DWI(+)**	**MED**	**Control**	**Group (A)**	**Phase (B)**	**A × B**
*n*	47	17	21	25	47	17	21	25			
Reading	50.0 ± 9.2	43.5 ± 12.2	45.4 ± 10.7	52.0 ± 8.8	46.0 ± 11.1^‡^	38.3 ± 15.1^†^^‡^	42.0 ± 10.2^‡^	49.8 ± 10.4	3.59^*^	27.22^***^	0.82
Verbal memory	43.6 ± 7.5^†^	44.9 ± 9.3	44.3 ± 9.0	49.7 ± 8.3	47.2 ± 10.8^‡^	45.0 ± 10.2	50.4 ± 11.7^‡^	53.8 ± 8.4^‡^	3.56^*^	16.12^***^	1.63
Visual memory	40.8 ± 11.6^†^	36.9 ± 14.1^†^	39.3 ± 10.6^†^	50.0 ± 8.3	43.4 ± 11.6^†^	39.6 ± 15.5^†^	46.4 ± 12.0^‡^	54.6 ± 9.1^‡^	7.41^***^	20.34^***^	1.34
Construction	47.0 ± 11.9	38.3 ± 18.6	45.6 ± 12.6	48.1 ± 12.4	48.2 ± 11.3	44.4 ± 15.1^‡^	47.9 ± 12.4	53.3 ± 8.9^‡^	2.45	8.14^**^	0.94
Visual motor	37.0 ± 10.8^†^^♦^	27.8 ± 13.5^†^	36.7 ± 8.8^†^	46.2 ± 9.1	38.5 ± 10.3^†^	32.5 ± 12.4^†^^‡^	40.1 ± 7.0^†^^‡^	49.6 ± 7.8^‡^	12.97^***^	15.55^***^	0.81
Design fluency	34.0 ± 18.0	32.8 ± 21.0	29.3 ± 19.7^†^	46.7 ± 20.1	39.1 ± 20.7^†^^‡^	33.3 ± 16.6^†^	30.5 ± 19.0^†^	56.7 ± 24.2^‡^	5.96^***^	6.77^*^	1.79
Word speed	38.7 ± 12.8^†^	43.5 ± 12.7	38.3 ± 10.4^†^	50.7 ± 9.5	38.6 ± 15.0^†^	40.8 ± 17.9	31.0 ± 15.0^†^^‡^	50.4 ± 9.1	6.91^***^	4.07^*^	1.97
Color speed	36.3 ± 16.0^†^	35.3 ± 18.3^†^	37.1 ± 12.3^†^	50.6 ± 8.1	38.5 ± 15.6^†^	32.3 ± 18.5^†^	33.2 ± 17.1^†^	49.6 ± 8.2	6.68^***^	1.68	2.18

*ANOVA, analysis of variance; DWI(–), absence of new ischemic lesions on diffusion-weighted imaging; DWI(+), presence of new ischemic lesions on diffusion-weighted imaging; MED, medication group*.

* *p < 0.05*,

** *p < 0.01, and*

*** *p < 0.001 main effect or interaction of ANOVA*.

† *p < 0.05 compared to the control group within each phase*.

♦ *p < 0.05 compared to the DWI+ group within each phase*.

‡ *p < 0.05 paired t-tests in each individual group between two phases*.

**Table 3:** Comparisons of cognitive domain performance before and after treatment by ANCOVA in Model I.

**Group**	**Pre-treatment phase**				**Post-treatment phase**				***F***		
	**DWI(–)**	**DWI(+)**	**MED**	**Control**	**DWI(–)**	**DWI(+)**	**MED**	**Control**	**Group (A)**	**Phase (B)**	**A × B**
*n*	47	17	21	25	47	17	21	25			
Reading	50.0 ± 9.2^♦^	43.5 ± 12.2^†^	45.4 ± 10.7	52.0 ± 8.8	46.0 ± 11.1^‡^	38.3 ± 15.1^†^^‡^	42.0 ± 10.2^‡^	49.8 ± 10.4	4.14^**^	4.93^*^	1.09
Verbal memory	43.6 ± 7.5^†^	44.9 ± 9.3	44.3 ± 9.0	49.7 ± 8.3	47.2 ± 10.8^‡^	45.0 ± 10.2^†^	50.4 ± 11.7^‡^	53.8 ± 8.4^‡^	3.17^*^	0.13	1.50
Visual memory	40.8 ± 11.6^†^	36.9 ± 14.1^†^	39.3 ± 10.6^†^	50.0 ± 8.3	43.4 ± 11.6^†^	39.6 ± 15.5^†^	46.4 ± 12.0^‡^	54.6 ± 9.1^‡^	6.96^***^	0.01	1.35
Construction	47.0 ± 11.9	38.3 ± 18.6	45.6 ± 12.6	48.1 ± 12.4	48.2 ± 11.3	44.4 ± 15.1^‡^	47.9 ± 12.4	53.3 ± 8.9^‡^	2.01	3.35	1.12
Visual motor	37.0 ± 10.8^†^♦	27.8 ± 13.5^†^	36.7 ± 8.8^†^	46.2 ± 9.1	38.5 ± 10.3^†^	32.5 ± 12.4^†^^‡^	40.1 ± 7.0^†^	49.6 ± 7.8^‡^	12.34^***^	0.78	0.82
Design fluency	34.0 ± 18.0^†^	32.8 ± 21.0	29.3 ± 19.7^†^	46.7 ± 20.1	39.1 ± 20.7^†^^‡^	33.3 ± 16.6^†^	30.5 ± 19.0^†^	56.7 ± 24.2^‡^	6.02^***^	1.19	1.63
Word speed	38.7 ± 12.8^†^	43.5 ± 12.7	38.3 ± 10.4^†^	50.7 ± 9.5	38.6 ± 15.0^†^	40.8 ± 17.9	31.0 ± 15.0^†^^‡^	50.4 ± 9.1	7.32^***^	0.65	1.67
Color speed	36.3 ± 16.0^†^	35.3 ± 18.3^†^	37.1 ± 12.3^†^	50.6 ± 8.1	38.5 ± 15.6^†^	32.3 ± 18.5^†^	33.2 ± 17.1^†^	49.6 ± 8.2	7.62^***^	0.06	2.15

*ANCOVA, analysis of covariance; DWI(–), absence of diffusion weighted ischemia; DWI(+), presence of diffusion weighted ischemia; MED, medication group*.

* *p < 0.05*,

** *p < 0.01, and*

*** *p < 0.001 main effect or interaction of ANCOVA after adjustment for age and education*.

† *p < 0.05 compared to the control group within each phase*.

♦ *p < 0.05 compared to the DWI+ group within each phase*.

‡ *p < 0.05 paired t-tests in each individual group between two phases*.

**Table 4:** Comparisons of cognitive domain performance before and after treatment by ANCOVA in Model II.

**Group**	**Pre-treatment phase**				**Post-treatment phase**				***F***		
	**DWI(–)**	**DWI(+)**	**MED**	**Control**	**DWI(–)**	**DWI(+)**	**MED**	**Control**	**Group (A)**	**Phase (B)**	**A × B**
*n*	47	17	21	25	47	17	21	25			
Reading	50.0 ± 9.2^♦^	43.5 ± 12.2^†^	45.4 ± 10.7	52.0 ± 8.8	46.0 ± 11.1^‡^	38.3 ± 15.1^†^^‡^	42.0 ± 10.2^‡^	49.8 ± 10.4	3.98^*^	4.49^*^	1.31
Verbal memory	43.6 ± 7.5^†^	44.9 ± 9.3	44.3 ± 9.0	49.7 ± 8.3	47.2 ± 10.8^†^^‡^	45.0 ± 10.2^†^	50.4 ± 11.7^‡^	53.8 ± 8.4^‡^	2.27	0.01	1.05
Visual memory	40.8 ± 11.6^†^	36.9 ± 14.1^†^	39.3 ± 10.6^†^	50.0 ± 8.3	43.4 ± 11.6^†^^‡^	39.6 ± 15.5^†^	46.4 ± 12.0^‡^	54.6 ± 9.1	5.00^**^	0.12	1.10
Construction	47.0 ± 11.9	38.3 ± 18.6	45.6 ± 12.6	48.1 ± 12.4	48.2 ± 11.3	44.4 ± 15.1^‡^	47.9 ± 12.4	53.3 ± 8.9^‡^	0.94	2.73	1.17
Visual motor	37.0 ± 10.8^†^♦	27.8 ± 13.5^†^	36.7 ± 8.8^†^	46.2 ± 9.1	38.5 ± 10.3^†^	32.5 ± 12.4^†^^‡^	40.1 ± 7.0^†^	49.6 ± 7.8^‡^	9.01^***^	0.77	0.81
Design fluency	34.0 ± 18.0^†^	32.8 ± 21.0	29.3 ± 19.7^†^	46.7 ± 20.1	39.1 ± 20.7^†^^‡^	33.3 ± 16.6^†^	30.5 ± 19.0^†^	56.7 ± 24.2^‡^	4.84^**^	0.77	1.30
Word speed	38.7 ± 12.8^†^	43.5 ± 12.7	38.3 ± 10.4^†^	50.7 ± 9.5	38.6 ± 15.0^†^	40.8 ± 17.9	31.0 ± 15.0^†^	50.4 ± 9.1	7.39^***^	1.08	1.83
Color speed	36.3 ± 16.0^†^	35.3 ± 18.3^†^	37.1 ± 12.3^†^	50.6 ± 8.1	38.5 ± 15.6^†^	32.3 ± 18.5^†^	33.2 ± 17.1^†^	49.6 ± 8.2	6.08^***^	0.03	2.16

*ANCOVA, analysis of covariance; DWI(–), absence of diffusion weighted ischemia; DWI(+), presence of diffusion weighted ischemia; MED, medication group*.

* *p < 0.05*,

** *p < 0.01, and*

*** *p < 0.001 main effect or interaction of ANCOVA after adjustment for age, education, and medial temporal atrophy*.

† *p < 0.05 compared to the control group within each phase*.

♦ *p < 0.05 compared to the DWI+ group within each phase*.

‡ *p < 0.05 paired t-tests in each individual group between two phases*.

**Table 5:** Comparisons of cognitive domain performance before and after treatment by ANCOVA in Model III.

**Group**	**Pre-treatment phase**				**Post-treatment phase**				***F***		
	**DWI(–)**	**DWI(+)**	**MED**	**Control**	**DWI(–)**	**DWI(+)**	**MED**	**Control**	**Group (A)**	**Phase (B)**	**A × B**
*n*	47	17	21	25	47	17	21	25			
Reading	50.0 ± 9.2^♦^	43.5 ± 12.2^†^	45.4 ± 10.7	52.0 ± 8.8	46.0 ± 11.1^‡^	38.3 ± 15.1^†^	42.0 ± 10.2^‡^	49.8 ± 10.4^‡^	3.07^*^	1.50	0.44
Verbal memory	43.6 ± 7.5^†^	44.9 ± 9.3	44.3 ± 9.0	49.7 ± 8.3	47.2 ± 10.8^‡^	45.0 ± 10.2^†^	50.4 ± 11.7^‡^	53.8 ± 8.4^‡^	0.35	0.11	0.76
Visual memory	40.8 ± 11.6^†^	36.9 ± 14.1^†^	39.3 ± 10.6^†^	50.0 ± 8.3	43.4 ± 11.6^†^	39.6 ± 15.5^†^	46.4 ± 12.0^‡^	54.6 ± 9.1^‡^	0.78	1.50	1.33
Construction	47.0 ± 11.9	38.3 ± 18.6	45.6 ± 12.6	48.1 ± 12.4	48.2 ± 11.3	44.4 ± 15.1^‡^	47.9 ± 12.4	53.3 ± 8.9^‡^	0.55	3.58	0.90
Visual motor	37.0 ± 10.8^†^♦	27.8 ± 13.5^†^	36.7 ± 8.8^†^	46.2 ± 9.1	38.5 ± 10.3^†^	32.5 ± 12.4^†^^‡^	40.1 ± 7.0^†^	49.6 ± 7.8^‡^	3.23^*^	0.75	0.69
Design fluency	34.0 ± 18.0^†^	32.8 ± 21.0	29.3 ± 19.7^†^	46.7 ± 20.1	39.1 ± 20.7^†^	33.3 ± 16.6^†^	30.5 ± 19.0^†^	56.7 ± 24.2^‡^	1.56	1.47	4.16^**^
Word speed	38.7 ± 12.8^†^	43.5 ± 12.7	38.3 ± 10.4^†^	50.7 ± 9.5	38.6 ± 15.0^†^	40.8 ± 17.9	31.0 ± 15.0^†^	50.4 ± 9.1	1.77	0.07	1.96
Color speed	36.3 ± 16.0^†^	35.3 ± 18.3^†^	37.1 ± 12.3^†^	50.6 ± 8.1	38.5 ± 15.6^†^	32.3 ± 18.5^†^	33.2 ± 17.1^†^	49.6 ± 8.2	1.38	0.69	2.36

*ANCOVA, analysis of covariance; DWI(–), absence of diffusion weighted ischemia; DWI(+), presence of diffusion weighted ischemia; MED, medication group*.

* p < 0.05 and ^**^p < 0.01 main effect or interaction of ANCOVA after adjustment for age, education, medial temporal atrophy, leukoaraiosis, infarct severity, and ipsilateral carotid stenosis severity.

† *p < 0.05 compared to the control group within each phase*.

♦ *p < 0.05 compared to the DWI+ group within each phase*.

‡ *p < 0.05 paired t-tests in each individual group between two phases*.

### Cognitive Changes Between Phases

#### Correlation Between Phases

**Figure 1:**
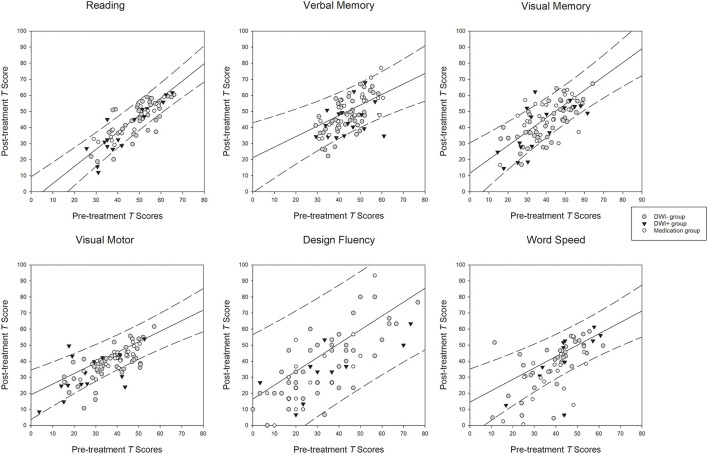
Relationships between the scores at the pre- and post-treatment phases. *Diagonal lines*: best-fit linear functions of the pre- and post-treatment scores in the control group. *Dashed lines*: 95% prediction intervals of the linear functions in the control group.

Only the tests that had main effect of phase and group difference on the two-factor ANOVA were plotted in Figure 1. The diagonal lines represent the best-fit linear functions and the dashed lines represent the 95% prediction intervals of the regression functions of the mean T scores of the specific cognitive domains between the two phases in the control group. Although there was a trend for improvement in the T scores of all cognitive domains across all groups (Table 2) and the correlations between the two phases were all positively significant (*rs* = >0.43, *p* = < 0.02; Supplementary Table 4), vast majority of the data points of all patients in each panel fell inside the prediction interval for the corresponding cognitive domain (Figure 1). Only one or two subjects in all patient groups showed reliable improvement in the post-treatment phase on each cognitive domain. In contrast, relatively more subjects in all patient groups showed significant deterioration on all cognitive domains.

#### Reliable Change Index

In order to account for practice effect resulting from repeated testing, we adopted the RCI derived from the controls to tally the proportions of reliable cognitive changes in each patient group (Table 6). We found the proportions of subjects in each patient group showing significant improvement or deterioration (±1 SD of the RCI) in each cognitive domain did not significantly differ.

**Table 6:** The proportions of patients with reliable changes in *T*-scores for each specific cognitive domain^a^.

	**DWI(–) group (*****n*** **=** **47)**		**DWI(+) group (*****n*** **=** **17)**		**MED group (*****n*** **=** **21)**		**χ^2^**	***p***
	**Improved**	**Deteriorated**	**Improved**	**Deteriorated**	**Improved**	**Deteriorated**		
Reading	10 (21)	15 (32)	1 (6)	6 (35)	2 (10)	3 (14)	6.75	0.15
Verbal memory	19 (21)	10 (21)	3 (18)	6 (35)	6 (29)	1 (5)	5.65	0.23
Visual memory	9 (19)	9 (43)	4 (24)	8 (47)	10 (48)	4 (19)	7.39	0.12
Visual motor	7 (15)	13 (28)	4 (24)	3 (18)	1 (5)	1 (5)	8.82	0.07
Design fluency	4 (9)	13 (28)	2 (12)	5 (30)	1 (5)	7 (33)	0.78	0.99
Word speed	5 (14)	8 (22)	2 (12)	2 (12)	2 (10)	8 (38)	3.86	0.43

*Only the cognitive domains that showed significant differences between pre- and post-treatment phases within groups are shown. Improved, 1 standard deviation above the mean of the reliable change index of the control group; deteriorated, 1 standard deviation below the mean of the reliable change index of the control group*.

*DWI(–), absence of diffusion weighted ischemia; DWI(+), presence of diffusion weighted ischemia; MED, medication group*.

a *Data is presented as n (%)*.

## Discussion

Carotid stenosis is a risk factor for cognitive impairment, and most of the studies have been focused on the perfusion restoration effects on cognitive function after carotid revascularization. Although a certain proportion of patients may suffer from cerebral silent ischemic lesions after carotid artery stenting, relatively few studies have addressed their cognitive sequalae. In this study, we adopted the MED group and the healthy volunteer group as the double control reference to gauge the cognitive changes after stenting. There were no differences in cognitive changes after treatment in the DWI(+), DWI(–) or the MED groups. On the other hand, the volunteer group was more likely to have better cognitive performance in the follow-up evaluation. These findings suggest cognitive performance may not change 6 months after carotid revascularization, irrespective of the presence of perfusion restoration or silent ischemic lesions, and practice effect should be taken into consideration when assessing the cognitive changes in patients with carotid stenosis.

Stroke risk is linearly associated with the carotid stenosis severity, and carotid revascularization by CEA or CAS has been suggested to prevent stroke occurrence in patients with significant carotid stenosis (Howard et al., 2021). Nevertheless, carotid artery stenting also harbors a peri-operative stroke and microemboli risk (Brott et al., 2016). Although no post-CAS stroke case was noted in our study, the percentage of post-CAS SILs was ~27%, which is in good agreement with previous findings that about 22–71% of CAS patients may have silent post-CAS DWI lesions (Witt et al., 2007; Tiemann et al., 2009; Capoccia et al., 2010; Huang et al., 2011, 2014a; Wasser et al., 2011; Zhou et al., 2012). Considering the high occurrence rate, it is important to identify whether these silent ischemic lesions would further lead to insidious cognitive decline. Some studies found post-CAS DWI lesions would induce cognitive decline on some single-domain cognitive tests, such as the MMSE and auditory verbal learning test (Capoccia et al., 2010; Huang et al., 2011; Zhou et al., 2012). However, the trend for DWI-related cognitive decline was not observed when further evaluating with more comprehensive neuropsychological tests involving multiple cognitive domains (Tiemann et al., 2009; Wasser et al., 2011). Such findings were compatible with our study results that the cognitive changes after CAS in patients with DWI(+) lesions were similar to those without DWI lesions as well as the MED group.

Although carotid artery stenting can restore cerebral hypoperfusion state, whether carotid revascularization is beneficial to cognitive improvement remains controversial (Porcu et al., 2020). In our study, the carotid stenosis severity was correlated with changes in executive function after carotid artery stenting. However, the cognitive impact of carotid artery stenting was not observed by the more conservative analysis, the RCI method. In Schroder and Wang's studies, MR perfusion scan was adopted to evaluate the relationship between hypoperfusion and cognitive changes after carotid stenting (Wang et al., 2017; Schroder et al., 2019). They found there was significant impact of revascularization on all perfusion parameters, but the correlation of perfusion improvement with carotid stenosis severity and cognitive changes was not significant. In another study restricting patient inclusion criteria to higher severity of carotid stenosis (>80%), cognitive benefits were observed in patients with MCA flow improvement (Whooley et al., 2020). The lack of consistent correlation between carotid revascularization and cognitive changes can be partly attributed to the intracranial collateral circulation system, which can dynamically provide compensatory flow to areas with hypoperfusion (Chuang et al., 2011; Shakur et al., 2014). Another possible mechanism is the neuroplasticity effect; neural hypoconnectivity ipsilateral to carotid stenosis side and hyperconnectivity in the contralateral hemisphere can be observed on functional MRI, and such asymmetric connectivity alteration gradually recovered to the normal condition after CAS (Huang et al., 2018). Therefore, neuroimaging markers with perfusion and neural connectivity information are important tools to evaluate the cognitive influence from carotid artery stenting in future studies.

Serial neuropsychological evaluation is useful for assessing cognitive changes in response to carotid revascularization. However, practice effect brought about by repeated testing is a formidable challenge to interpret neuropsychological results, because it has proved difficult to dissociate the practice effect from a real change in cognition, particularly when the same testing materials were administered repeatedly (Bartels et al., 2010; Rao, 2014). To control for practice effect in our study, we recruited the MED group and healthy volunteers as the reference groups, and both groups received the same repeated cognitive testing protocols as the CAS groups. Interestingly, the healthy subjects showed cognitive improvements in nearly all cognitive domains, suggesting the practice effect could still exist even when the cognitive testing was repeated 6 months apart. The cognitive changes in healthy volunteers were further epitomized by the best-fitted linear functions depicted in Figure 1. Although all patient groups also demonstrated upward trend of the linear relationships between the pre- and post-treatment scores (Table 2), relatively few subjects in the patient groups falling above the upper limits of the predictive intervals of the linear functions of the volunteers in all cognitive domains, but there were slightly more clinical subjects falling beyond the lower limits of the predictive intervals. These observations suggest there was no solid evidence for real changes in most cognitive domains between pre- and post-treatment in subjects with carotid artery stenosis.

In addition to using regression approach in examining the practice effects, we also utilized the reliable change index approach to measure the cognitive changes, which has been widely used in neuropsychological literature for controlling the practice effects of repeated testing (Tuokko and Smart, 2018). In agreement with the regression approach, the proportions of subjects showing reliable changes (improvement or decline) did not significantly differ between the DWI(–), DWI(+), and MED groups when using the data of volunteers as a reference. This lends further support for the notion that neither carotid revascularization nor silent ischemic lesions contribute to cognitive function after receiving carotid artery stenting.

The general lack of reliable cognitive changes following carotid revascularization in this study raises the doubt that whether the cognitive impairments detected in subjects with carotid artery stenosis at the pre- and post-treatment phases could be directly attributed to cerebral hypoperfusion brought about by carotid stenosis. Indeed, the two-way ANOVA revealed that although there were significant main phase effects for most specific cognitive domains, these main effects of phase diminished after using age, education, and MTA score as covariates. In addition, all these factors were also distinctly and significantly correlated with the baseline cognitive performance depending on which cognitive domains were in question in this study. This is consistent with the suggestion that age, education and MTA might play a pivotal role in moderating cognitive performance in most intervention studies, including the present one (Lezak et al., 2012). Compared to healthy volunteers, subjects with carotid artery stenosis tended to be older with lower educational attainments and have more severe MTA scores in this study. It is quite possible that the presence of these risk factors could impede the improvement in cognition, if any, following carotid revascularization. However, the sample size of this study was relatively small, and the statistical results should be interpreted cautiously when applying to general CS patients. Clearly, further studies with more subjects to explore the possible moderating role of these factors in the relationships between carotid stenosis and cognition are deemed necessary.

There were some limitations in our study. Firstly, the study protocol did not include perfusion and neural connectivity parameters. Considering the great variation of collateral flow system and autoregulation mechanism, patients with significant carotid stenosis should be further grouped by their perfusion states in order to elucidate the interaction between hypoperfusion, altered neural connectivity and related cognitive impairment. Secondly, not all acute DWI lesions would evolve to cavitation in the chronic stage (Koch et al., 2011), and follow-up image was not done for the peri-CAS silent ischemic lesions in our study. The cognitive influence of DWI(+) lesions could be further stratified depending on whether they result in permanent structure injury. Furthermore, cognitive trajectories after vascular events are dynamic and versatile. The cognitive changes 6 months after CAS in this study could merely represent a tentative impression of the cognitive influence from SILs. Therefore, long-term follow-up with multiple assessment sessions to delineate the cognitive trajectories can be of immense value, and would be helpful for planning treatment strategies for patients with carotid stenosis. Finally, the sample size was relatively small, and the statistical results may be underpowered to demonstrate the cognitive influence from silent ischemic lesions.

## Conclusion

Carotid stenosis can lead to vascular cognitive impairment through multiple mechanisms. Although the cognitive sequalae from procedure-related silent ischemic lesion was not significant, the overall cognitive benefit from carotid artery stenting still requires further investigation.

## Data Availability Statement

The datasets presented in this article are not readily available because the used consent does not allow for the public sharing of the data. Requests to access the datasets should be directed to thlee@adm.cgmh.org.tw.

## Ethics Statement

The studies involving human participants were reviewed and approved by the Institutional Review Board of Chang Gung Memorial Hospital. The patients/participants provided their written informed consent to participate in this study.

## Author Contributions

K-LH wrote the initial draft, took part in the data collection and analysis, and scientific interpretation of data. M-YH conceptualized the study design, wrote a portion of the draft, conducted the cognitive evaluation, took part in data analysis, and critical review of the manuscript. Y-JC, C-HC, C-HL, and H-CW performed the data collection, neurological examination, and scientific interpretation of data. T-YC performed the MRI analysis, took part in data interpretation, and critical review of the manuscript. T-HL conceptualized the study design, took part in critical review of the manuscript, and edited the manuscript. All authors critically reviewed the manuscript and approved the final version for publication.

## Funding

This research was funded by Ministry of Science and Technology, Taiwan (MOST 110-2314-B-182A-073-MY3 and MOST 107-2410-H-182-008-MY3) and Research Fund of Chang Gung Memorial Hospital (CMRPG3J1193, CPRPG3H0013, CMRPG3F2183, and BMRP611).

## Conflict of Interest

The authors declare that the research was conducted in the absence of any commercial or financial relationships that could be construed as a potential conflict of interest.

## Publisher's Note

All claims expressed in this article are solely those of the authors and do not necessarily represent those of their affiliated organizations, or those of the publisher, the editors and the reviewers. Any product that may be evaluated in this article, or claim that may be made by its manufacturer, is not guaranteed or endorsed by the publisher.

## References

[B1] Bartels C. Wegrzyn M. Wiedl A. Ackermann V. Ehrenreich H. (2010). Practice effects in healthy adults: a longitudinal study on frequent repetitive cognitive testing. BMC Neurosci. 11:118. 10.1186/1471-2202-11-118 20846444 PMC2955045

[B2] Bonati L. H. Jongen L. M. Haller S. Flach H. Z. Dobson J. Nederkoorn P. J.. (2010). New ischaemic brain lesions on MRI after stenting or endarterectomy for symptomatic carotid stenosis: a substudy of the International Carotid Stenting Study (ICSS). Lancet Neurol. 9, 353–362. 10.1016/S1474-4422(10)70057-0 20189458

[B3] Brott T. G. Howard G. Roubin G. S. Meschia J. F. Mackey A. Brooks W.. (2016). Long-term results of stenting versus endarterectomy for carotid-artery stenosis. N. Engl. J. Med. 374, 1021–1031. 10.1056/NEJMoa1505215 26890472 PMC4874663

[B4] Capoccia L. Speziale F. Gazzetti M. Mariani P. Rizzo A. Mansour W.. (2010). Comparative study on carotid revascularization (endarterectomy vs stenting) using markers of cellular brain injury, neuropsychometric tests, and diffusion-weighted magnetic resonance imaging. J. Vasc. Surg. 51, 584–591, 591.e1–3; discussion: 592. 10.1016/j.jvs.2009.10.079 20045614

[B5] Chelune G. J. Naugle R. I. Lüders H. Sedlak J. Awad I. A. J. N. (1993). Individual change after epilepsy surgery: practice effects and base-rate information. Neuropsychology 7:41. 10.1037/0894-4105.7.1.41

[B6] Chen Y.-J. Ho M.-Y. Chen K.-J. Hsu C.-F. Ryu S.-J. (2009). Estimation of premorbid general fluid intelligence using traditional Chinese reading performance. Psychiatry Clin. Neurosci. 63, 500–507. 10.1111/j.1440-1819.2009.01970.x 19460119

[B7] Chuang Y.-M. Chang Y.-J. Chang C.-H. Huang K.-L. Chang T.-Y. Wu T.-C.. (2011). Correlation between the flow pattern of the circle of Willis and segmental perfusion asymmetry after carotid artery revascularization. Eur. J. Neurol. 18, 1132–1138. 10.1111/j.1468-1331.2010.03344.x 21299732

[B8] Howard D. P. J. Gaziano L. Rothwell P. M. (2021). Risk of stroke in relation to degree of asymptomatic carotid stenosis: a population-based cohort study, systematic review, and meta-analysis. Lancet Neurol. 20, 193–202. 10.1016/S1474-4422(20)30484-1 33609477 PMC7889579

[B9] Huang H. S. (2001). Chinese Graded Word Reading Test [in Chinese]. Taipei: Psychological Publishing.

[B10] Huang K.-L. Chang T.-Y. Chang C.-H. Liu H.-L. Chang Y.-J. Liu C.-H.. (2014a). Relationships between ophthalmic artery flow direction and cognitive performance in patients with unilateral carotid artery stenosis. J. Neurol. Sci. 336, 184–190. 10.1016/j.jns.2013.10.037 24211063

[B11] Huang K.-L. Chang T.-Y. Ho M.-Y. Chen W.-H. Yeh M.-Y. Chang Y.-J.. (2018). The correlation of asymmetrical functional connectivity with cognition and reperfusion in carotid stenosis patients. Neuroimage Clin. 20, 476–484. 10.1016/j.nicl.2018.08.011 30128286 PMC6098231

[B12] Huang K.-L. Chang Y.-J. Chang C.-H. Chang T.-Y. Liu C.-H. Hsieh I. C.. (2014b). Impact of coexisting coronary artery disease on the occurrence of cerebral ischemic lesions after carotid stenting. PLoS ONE 9:e94280. 10.1371/journal.pone.0094280 24732408 PMC3986076

[B13] Huang K.-L. Ho M.-Y. Chang C.-H. Ryu S.-J. Wong H.-F. Hsieh I.-C.. (2011). The impact of silent ischemic lesions on cognition following carotid artery stenting. Eur. Neurol. 66, 351–358. 10.1159/000332614 22123044

[B14] Kastrup A. Gröschel K. Krapf H. Brehm B. R. Dichgans J. Schulz J.. (2003). Early outcome of carotid angioplasty and stenting with and without cerebral protection devices: a systematic review of the literature. Stroke 34, 813–819. 10.1161/01.STR.0000058160.53040.5F 12624315

[B15] Koch S. McClendon M. S. Bhatia R. (2011). Imaging evolution of acute lacunar infarction: leukoariosis or lacune? Neurology 77, 1091–1095. 10.1212/WNL.0b013e31822e1470 21880998

[B16] Laza C. Popescu B. O. Popa M. Roceanu A. M. Tiu C. Antochi F. A.. (2013). Microemboli detection in patients with carotid artery stenting – a potential marker for future cognitive impairment? J. Neurol. Sci. 326, 96–99. 10.1016/j.jns.2013.01.025 23403326

[B17] Lee T.-H. Huang K.-L. Chang T.-Y. Ho M.-Y. Wey S.-P. Hsieh C.-J.. (2017). Early-phase 18F-AV-45 PET imaging can detect crossed cerebellar diaschisis following carotid artery stenosis and cerebral hypoperfusion. Curr. Neurovasc. Res. 14, 258–265. 10.2174/1567202614666170621102101 28637413

[B18] Lee T. H. Chang C. H. Chang Y. J. Chang K. C. Chung J. (2011). Establishment of electronic chart-based stroke registry system in a medical system in Taiwan. J. Formos. Med. Assoc. 110, 543–547. 10.1016/S0929-6646(11)60081-8 21783024

[B19] Lezak M. D. Howieson D. B. Bigler E. D. Tranel D. (2012). Neuropsychological Assessment. New York, NY: Oxford University Press.

[B20] Madigan J. B. Wilcock D. M. Hainsworth A. H. (2016). Vascular contributions to cognitive impairment and dementia. Stroke 47, 1953–1959. 10.1161/STROKEAHA.116.012066 27301939 PMC4927375

[B21] Moroni F. Ammirati E. Magnoni M. D'Ascenzo F. Anselmino M. Anzalone N.. (2016). Carotid atherosclerosis, silent ischemic brain damage and brain atrophy: a systematic review and meta-analysis. Int. J. Cardiol. 223, 681–687. 10.1016/j.ijcard.2016.08.234 27568989

[B22] Paraskevas K. I. Lazaridis C. Andrews C. M. Veith F. J. Giannoukas A. D. (2014). Comparison of cognitive function after carotid artery stenting versus carotid endarterectomy. Eur. J. Vasc. Endovasc. Surg. 47, 221–231. 10.1016/j.ejvs.2013.11.006 24393665

[B23] Porcu M. Cocco L. Saloner D. Suri J. S. Montisci R. Carriero A.. (2020). Extracranial carotid artery stenosis: the effects on brain and cognition with a focus on resting-state functional connectivity. J. Neuroimaging 30, 736–745. 10.1111/jon.12777 32866351

[B24] Rao S. (2014). Analysis of practice effects across cognitive domains in mild cognitive impairment (master of rehabilitation counseling). The University of Texas Southwestern Medical Center, Dallas, TX, United States.32594886

[B25] Scheltens P. van de Pol L. (2012). Atrophy of medial temporal lobes on MRI in “probable” Alzheimer's disease and normal ageing: diagnostic value and neuropsychological correlates. J. Neurol. Neurosurg. Psychiatry 83, 1038–1040. 10.1136/jnnp-2012-302562 22566596

[B26] Schroder J. Heinze M. Gunther M. Cheng B. Nickel A. Schroder T.. (2019). Dynamics of brain perfusion and cognitive performance in revascularization of carotid artery stenosis. Neuroimage Clin. 22:101779. 10.1016/j.nicl.2019.101779 30903966 PMC6431743

[B27] Shakur S. F. Hrbac T. Alaraj A. Du X. Aletich V. A. Charbel F. T.. (2014). Effects of extracranial carotid stenosis on intracranial blood flow. Stroke 45, 3427–3429. 10.1161/STROKEAHA.114.006622 25228258

[B28] Sztriha L. K. Nemeth D. Sefcsik T. Vecsei L. (2009). Carotid stenosis and the cognitive function. J. Neurol. Sci. 283, 36–40. 10.1016/j.jns.2009.02.307 19269651

[B29] Tiemann L. Reidt J. H. Esposito L. Sander D. Theiss W. Poppert H. J. P. O. (2009). Neuropsychological sequelae of carotid angioplasty with stent placement: correlation with ischemic lesions in diffusion weighted imaging. PLoS ONE 4:e7001. 10.1371/journal.pone.0007001 19746158 PMC2734991

[B30] Tuokko H. A. Smart C. M. (2018). Neuropsychology of Cognitive Decline: A Developmental Approach to Assessment and Intervention. New York: Guilford Press.

[B31] Wahlund L. O. Barkhof F. Fazekas F. Bronge L. Augustin M. Sjogren M.. (2001). A new rating scale for age-related white matter changes applicable to MRI and CT. Stroke 32, 1318–1322. 10.1161/01.STR.32.6.1318 11387493

[B32] Wallin A. Roman G. C. Esiri M. Kettunen P. Svensson J. Paraskevas G. P.. (2018). Update on vascular cognitive impairment associated with subcortical small-vessel disease. J. Alzheimers Dis. 62, 1417–1441. 10.3233/JAD-170803 29562536 PMC5870030

[B33] Wang T. Sun D. Liu Y. Mei B. Li H. Zhang S.. (2017). The impact of carotid artery stenting on cerebral perfusion, functional connectivity, and cognition in severe asymptomatic carotid stenosis patients. Front. Neurol. 8:403. 10.3389/fneur.2017.00403 28848495 PMC5552726

[B34] Wasser K. Pilgram-Pastor S. M. Schnaudigel S. Stojanovic T. Schmidt H. Knauf J.. (2011). New brain lesions after carotid revascularization are not associated with cognitive performance. J. Vasc. Surg. 53, 61–70. 10.1016/j.jvs.2010.07.061 20875716

[B35] Whooley J. L. David B. C. Woo H. H. Hoh B. L. Raftery K. B. Hussain Siddiqui A.. (2020). Carotid revascularization and its effect on cognitive function: a prospective nonrandomized multicenter clinical study. J. Stroke Cerebrovasc. Dis. 29:104702. 10.1016/j.jstrokecerebrovasdis.2020.104702 32107155

[B36] Witt K. Börsch K. Daniels C. Walluscheck K. Alfke K. Jansen O.. (2007). Neuropsychological consequences of endarterectomy and endovascular angioplasty with stent placement for treatment of symptomatic carotid stenosis. J. Neurol. 254, 1524–1532. 10.1007/s00415-007-0576-x 17657403

[B37] Zhou W. Hitchner E. Gillis K. Sun L. Floyd R. Lane B.. (2012). Prospective neurocognitive evaluation of patients undergoing carotid interventions. J. Vasc. Surg. 56, 1571–1578. 10.1016/j.jvs.2012.05.092 22889720 PMC3508143

