# Supplementary material for: Cognitive Sequelae of Silent Ischemic Lesions Following Carotid Artery Stenting: Possible Role of Age-Related Moderation
Source: Front Aging Neurosci. 2022 Jan 12;13:732617. doi: 10.3389/fnagi.2021.732617 (PMC8789655; doi:10.3389/fnagi.2021.732617)
Supplement: Supplementary file 1 [file Data_Sheet_1.docx]

| Supplementary Table 1. Measures of neuropsychological tests corresponding to each specific cognitive domain. | |
| --- | --- |
| Reading | Chinese Graded Word Reading Test |
| Verbal Memory | The Immediate Total Recall, Free Delay Recall, and Recognition Discriminability of the CVLT-2 |
| Visual Recall | The Immediate Total Recall, Free Delay Recall, Recognition Discriminability of the BVMT-R; The Position Discrimination of the VOSP |
| Construction | Benton 3-dimensional Constructional Praxis Test |
| Visuo-motor | The Purdue Pegboard Test for both hands; Number Location of the VOSP |
| Design Fluency | Total Correct of the Filled, Empty and Switch trials of the Design Fluency Test the D-KEFS |
| Verbal Speed | Mean reaction times of all trials of the Word versions of the Computerized Stroop Test |
| Visual Speed | Mean reaction times of all trials of the Color versions of the Computerized Stroop Test; Color Trail Tests |
| *BVMT-R, Brief Visual Memory Test-Revised; CVLT-2, California Verbal Learning Test (2^nd^ ed.); D-KEFS, Delis-Kaplan Executive Function System; VOSP, Visual Object and Spatial Perception Battery.* | |

| Supplementary Table 2. Correlation of baseline cognitive performance with demographic data and image findings in patients with carotid stenosis. | | | | | | | |
| --- | --- | --- | --- | --- | --- | --- | --- |
| Baseline cognition | Age | Education | MTA score | Ipsilateral stenosis | Contralateral stenosis | Infarct score | Leukoaraiosis |
| Reading | -0.17 | 0.58^***^ | -0.10 | -0.03 | 0.19 | -0.10 | -0.11 |
| Verbal memory | -0.05 | 0.23^*^ | 000 | -0.02 | -0.12 | -0.05 | -0.09 |
| Visual memory | -0.08 | 0.30^**^ | -0.12 | -0.08 | 0.07 | -0.14 | -0.20 |
| Construction | -0.14 | 0.26^*^ | -0.34^**^ | -0.01 | 0.03 | -0.16 | -0.33^*^ |
| Visual motor | -0.03 | 0.07 | -0.31^**^ | -0.02 | 0.01 | -0.25^*^ | -0.29^*^ |
| Design fluency | 0.06 | 0.33^**^ | -0.16 | -0.24^*^ | -0.18 | -0.17 | -0.19 |
| Word speed | -0.27^*^ | 0.17 | -0.15 | -0.08 | -0.18 | -0.04 | -0.05 |
| Color speed | -0.49 | 0.31^**^ | -0.42^***^ | 0.08 | -0.11 | -0.17 | -0.2 |
| *MTA, medial temporal atrophy.*  **p <0.05, **p<0.01, ***p<0.001.* | | | | | | | |

| Supplementary Table 3. Correlation coefficients of cognitive changes with demographic data and image findings in patients receiving carotid artery stenting. | | | | | | | |
| --- | --- | --- | --- | --- | --- | --- | --- |
| Cognitive changes | Age | Education | MTA score | Ipsilateral stenosis | Contralateral stenosis | Infarct score | Leukoaraiosis |
| Reading | 0.26 | -0.02 | 0.12 | -0.10 | 0.01 | -0.13 | -0.11 |
| Verbal memory | -0.13 | 0.19 | -0.22 | -0.14 | 0.10 | -0.20 | -0.20 |
| Visual memory | 0.03 | -0.08 | -0.34^**^ | 0.03 | -0.10 | -0.16 | 0.03 |
| Construction | -0.12 | -0.15 | -0.06 | -0.11 | -0.05 | 0.01 | 0.12 |
| Visual motor | 0.02 | -0.09 | 0.02 | -0.02 | 0.00 | 0.10 | 0.12 |
| Design fluency | -0.23 | 0.07 | -0.30^*^ | 0.32^*^ | -0.09 | -0.17 | -0.03 |
| Word speed | -0.1 | 0.29 | 0.13 | -0.08 | 0.30^*^ | 0.30^*^ | 0.09 |
| Color speed | 0.00 | -0.03 | 0.01 | -0.08 | 0.20 | 0.19 | 0.18 |
| *MTA, medial temporal atrophy.*  **p <0.05, **p<0.01, ***p<0.001.* | | | | | | | |

| Supplementary Table 4. Regression coefficients of the linear relations between pre- and post-treatment phases on different cognitive domains. | | | | | | | | | | | | | | | |
| --- | --- | --- | --- | --- | --- | --- | --- | --- | --- | --- | --- | --- | --- | --- | --- |
|  | DWI(-) | | |  | DWI(+) | | |  | MED | | |  | Control | | |
|  | *n*=47 | | |  | *n*=17 | | |  | *n*=21 | | |  | *n*=25 | | |
| Cognitive domains | B | SE | 95%CI |  | B | SE | 95%CI |  | B | SE | 95%CI |  | B | SE | 95%CI |
| Reading | 0.89^***^ | 0.14 | [0.61, 1.16] |  | 1.11^***^ | 0.14 | [0.82, 1.41] |  | 0.89^***^ | 0.10 | [0.67, 1.10] |  | 1.07^***^ | 0.11 | [0.84, 1.29] |
| Verbal memory | 0.91^***^ | 0.17 | [0.57, 1.24] |  | 0.36 | 0.27 | [-0.21, 0.94] |  | 0.95^***^ | 0.17 | [0.60, 1.30] |  | 0.66^**^ | 0.16 | [0.32, 0.99] |
| Visual memory | 0.72^**^ | 0.11 | [0.51, 0.93] |  | 0.79^**^ | 0.20 | [0.37, 1.21] |  | 0.53^*^ | 0.21 | [0.09, 0.98] |  | 0.86^***^ | 0.14 | [0.57, 1.15] |
| Construction | 0.39^**^ | 0.13 | [0.12, 0.65] |  | 0.59^**^ | 0.15 | [0.27, 0.90] |  | 0.58^**^ | 0.18 | [0.21, 0.95] |  | 0.21 | 0.14 | [-0.09, 0.51] |
| Visual motor | 0.66^***^ | 0.10 | [0.45, 0.86] |  | 0.51^*^ | 0.21 | [0.07, 0.95] |  | 0.69^***^ | 0.08 | [0.52, 0.86] |  | 0.66^***^ | 0.12 | [0.42, 0.90] |
| Design fluency | 0.86^***^ | 0.18 | [0.62, 1.09] |  | 0.60^**^ | 0.16 | [0.24, 0.97] |  | 0.91^***^ | 0.17 | [0.56, 1.26] |  | 0.86^***^ | 0.18 | [0.50, 1.23] |
| Word speed | 0.74^***^ | 0.16 | [0.42, 1.06] |  | 1.00^*^ | 0.33 | [0.25, 1.75] |  | 0.92^**^ | 0.28 | [0.34, 1.51] |  | 0.71^***^ | 0.15 | [0.40, 1.02] |
| Color speed | 0.80^***^ | 0.09 | [0.61, 0.99] |  | 0.79^**^ | 0.21 | [0.31, 1.27] |  | 1.13^***^ | 0.19 | [0.73, 1.53] |  | 0.71^***^ | 0.15 | [0.39, 1.02] |
| *The regression analyses were performed by regressing the T-scores of the post-treatment phase on those of the pre-treatment phase for each group. 95%CI=95% confidence interval; DWI(-), absence of diffusion weighted ischemia; DWI(+), presence of diffusion weighted ischemia; MED, medication group.*  *^***^p<0.0001; ^**^p<0.01; ^*^p<0.05.* | | | | | | | | | | | | | | | |
